# Colonic Diverticulitis Complicated by Stenosis Causing Bowel Obstruction

**DOI:** 10.7759/cureus.30956

**Published:** 2022-11-01

**Authors:** Teruaki Inoue

**Affiliations:** 1 Department of Internal Medicine, Fujinomiya City General Hospital, Fujinomiya, JPN

**Keywords:** chronic inflammation, colectomy, bowel obstruction, stenosis, diverticulitis

## Abstract

Colonic diverticulitis is one of the most common gastrointestinal diseases. There are several complications in colonic diverticulitis, such as stenosis, perforation, and abscess. Stenosis is a rare complication and can cause bowel obstruction. We report a case of colonic diverticulitis complicated by stenosis causing bowel obstruction.

A 66-year-old Japanese man was referred to our hospital for abdominal pain. Computed tomography (CT) scans showed the presence of diverticula, concentric wall thickening, and pericolic fat stranding in the descending colon. He was diagnosed with descending colon diverticulitis. His abdominal pain improved with fasting and intravenous antibiotics. However, after three months, diverticulitis complicated by stenosis occurred in the descending colon. The stenosis was severe and was treated with left hemicolectomy. Histologic examination revealed diverticula, chronic inflammation, and fibrosis in the stenosis, with no malignancy.

Stenosis caused by colonic diverticulitis can cause bowel obstruction. Conservative treatment may lead to recurrence, and surgical treatment is preferable.

## Introduction

Colonic diverticulitis is a common gastrointestinal disease and its incidence increases with age. It occurs when the diverticulum becomes inflamed, with the lifelong risk of diverticulitis reported to range from 10% to 25% in people with diverticula [1]. The main symptoms include sharp pain, vomiting, and fever, which can be complicated by stenosis, perforation, and abscess. Stenosis is a rare complication, and only 0.09% of patients with colonic diverticulosis develop diverticulitis, resulting in stenosis [2]. Stenosis can cause bowel obstruction and early intervention is required. We report a case of colonic diverticulitis complicated by stenosis causing bowel obstruction.

## Case presentation

**Figure 1:**
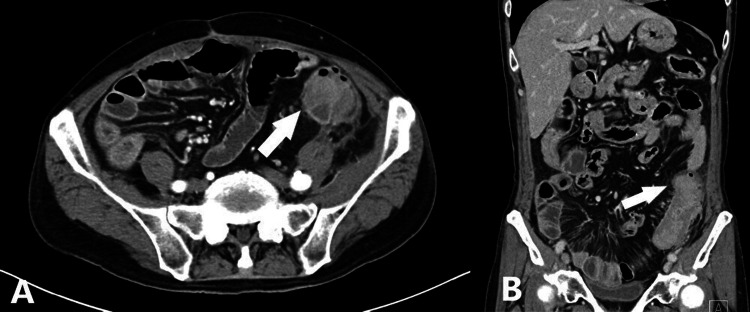
Abdominal contrast-enhanced CT scans, axial view (A) and coronal view (B) Diverticula, concentric wall thickening, and pericolic fat stranding were observed in the descending colon (white arrow).

A 66-year-old Japanese man was referred to our hospital for abdominal pain that lasted a day. He had a past medical history of cecal perforation caused by diverticulitis and took no significant medication. Upon examination, his vital signs were normal. Abdominal examination revealed left lower quadrant pain. A blood test showed an elevated C-reactive protein (CRP) of 18.42 mg/dL. Computed tomography (CT) scans showed the presence of diverticula, concentric wall thickening, and pericolic fat stranding in the descending colon (Figures 1A, 1B).

**Figure 2:**
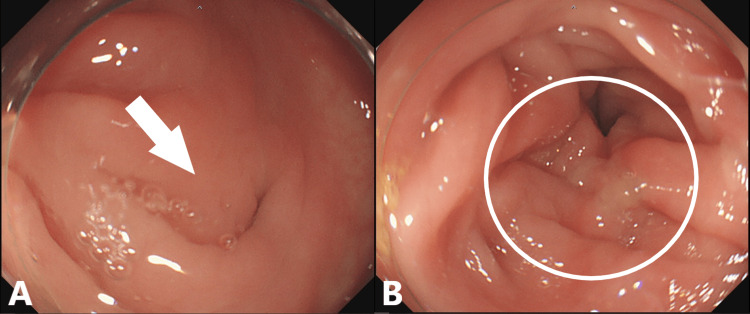
Colonoscopy finding The colonoscopy showed diverticula (A, white arrow) and wall edema (B, encircled lesion) in the descending colon.

He was consequently diagnosed with descending colon diverticulitis and was admitted to our hospital on the same day. He was kept nil by mouth and treated with appropriate antibiotics, cefmetazole sodium, and analgesia. His abdominal pain improved and a blood test showed a decrease in CRP levels. After starting oral intake, he had no recurrent symptoms. On the tenth hospital day, he was discharged from the hospital. After discharge, he underwent a colonoscopy to rule out colorectal cancer. The colonoscopy revealed diverticulum and wall edema in the descending colon but no tumor (Figures 2A, 2B).

**Figure 3:**
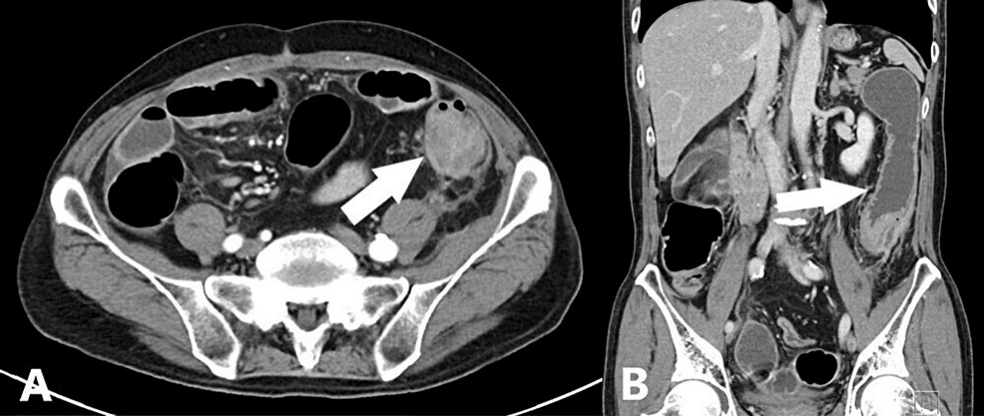
Abdominal contrast-enhanced CT scans, axial view (A) and coronal view (B) Wall edema (A, white arrow) and dilatation of the oral side (B, white arrow) were observed in the descending colon.

A tumor was not found during routine follow-up. But suddenly, he developed abdominal pain and came to our emergency room (ER) after three months. CT revealed a relapse of diverticulitis complicated by bowel obstruction in the descending colon (Figures 3A, 3B), and the patient was admitted to our hospital again.

**Figure 4:**
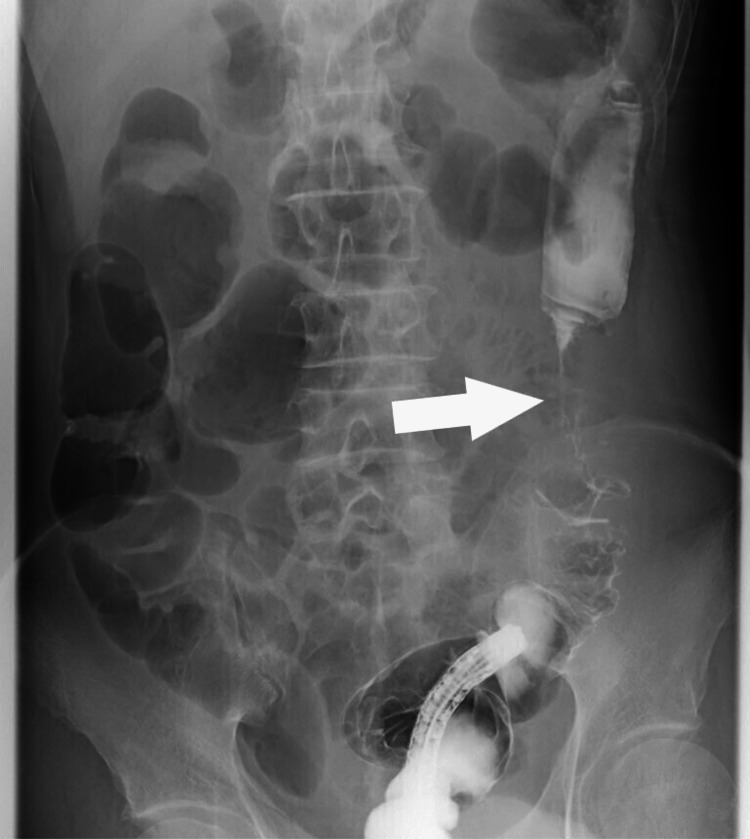
Abdominal X-ray A Gastrografin enema showed severe stenosis in the descending colon (white arrow).

A Gastrografin enema study showed a 6 cm-long segmental stenosis in the descending colon (Figure 4).

**Figure 5:**
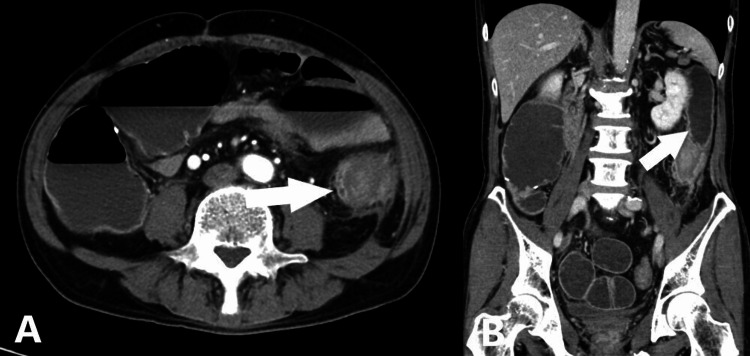
Abdominal contrast-enhanced CT scans, axial view (A) and coronal view (B) Wall edema (A, white arrow) and dilatation of the oral side (B, white arrow) were observed in the descending colon.

During evaluation and close follow-up, he was diagnosed with diverticulitis complicated by stenosis in the descending colon. Fasting and antibiotics improved bowel obstruction but after two months, bowel obstruction caused by diverticulitis relapsed (Figures 5A, 5B).

**Figure 6:**
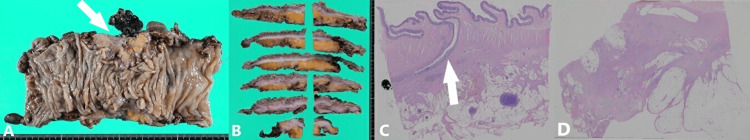
Resected specimen (A, B) and pathological image (C, D) Wall thickening and fibrosis were observed in the resected specimen, with no evidence of malignancy (A, B, white arrow). A pathological image of the resected specimen showed some diverticula (C, white arrow) and fibrosis (D) in the descending colon.

He was treated with a transanal ileus tube. Following this course, to improve bowel obstruction, a descending colectomy was performed. Histologic examination revealed diverticula, chronic inflammation, and fibrosis in the stenosis, with no malignancy (Figure 6).

After surgery, the bowel obstruction and symptoms improved, and the patient was able to resume oral intake. At present, he has no recurrence of bowel obstruction and diverticulitis.

## Discussion

A colonic diverticulum is an outpouching of the wall of the colon, which forms a sac. According to modern studies, the risk of developing colonic diverticulitis is less than 5% [1]. The rate of acute uncomplicated colonic diverticulitis is approximately 70%, and it can be treated conservatively [3]. Complicated colonic diverticulitis sometimes requires surgical treatment. Stenosis is a rare complication of colonic diverticulitis and can cause bowel obstruction [2]. Yoshida et al. reported a case of ascending colon stenosis caused by chronic diverticulitis [4]. Stenosis is caused by chronic diverticulitis, which leads to fibrosis obstruction of the colonic lumen [2]. It is sometimes difficult to distinguish between benign and malignant colorectal obstruction. However, identifying the cause of obstruction is very important because malignant stenosis requires early diagnosis and intervention. In Chintapalli’s study, pericolonic inflammation and segment involvement greater than 10 cm on computed tomography (CT) scans were significant findings for colonic diverticulitis [5]. In contrast, luminal mass and pericolonic lymph nodes were significant findings for colon cancer [5]. In addition, based on recent studies, Nishiyama et al. reported that magnetic resonance imaging diffusion-weighted imaging (MRI-DWI) is useful when distinguishing between colon cancer and inflammation is difficult [6]. In our case, CT scans showed pericolonic inflammation and segment involvement greater than 10 cm (Figure 1), and the cause of stenosis was thought to be inflammation.

Stenosis can cause severe distension of the colon, resulting in continuous ischemia of all bowel wall layers and necrosis of the colon [2]. Therefore, when there is severe distension, decompression with an ileus tube or early surgical treatment should be conducted. Our patient had severe distention of the colon caused by stenosis and was treated with an ileus tube. There have been reports that a self-expanding metal stent (SEMS) can be used for treating stenosis caused by diverticular disease [7,8]. Ohata et al. reported a case in which SEMS was inserted for stenosis due to colonic diverticulitis [8]. However, because of the considerable risk of complications, if a SEMS is used for diverticular disease, bowel resection should be performed at the site of stent insertion within a month [9]. Our patient might have been indicated for treatment with SEMS because we were planning a descending colectomy for him.

As our case showed, once bowel obstruction caused by diverticulitis occurs, it may occur again, even if conservative treatment improves it. In addition, Tehranian et al. reported that the incidence of colorectal cancer and advanced adenoma was higher in patients with diverticulitis as compared with subjects undergoing screening colonoscopy [10]. Hence, when severe stenosis caused by colonic diverticulitis occurs, we should consider surgical treatment such as colectomy. However, since immediate definitive surgery has several complications such as anastomotic leakage, initial decompression with a transanal ileus tube or SEMS followed by surgery is preferable when stenosis occurs.

## Conclusions

In summary, we have presented a case of colonic diverticulitis complicated by stenosis. Stenosis caused by colonic diverticulitis can cause large bowel obstruction and impending rupture. Conservative treatment may lead to recurrence, and early surgical treatment is the preferable basis to prevent further complications.

## References

[1] Strate LL, Morris AM (2019). Epidemiology, pathophysiology, and treatment of diverticulitis. Gastroenterology.

[2] Antonopoulos P, Almyroudi M, Kolonia V, Kouris S, Troumpoukis N, Economou N (2013). Toxic megacolon and acute ischemia of the colon due to sigmoid stenosis related to diverticulitis. Case Rep Gastroenterol.

[3] Andersen JC, Bundgaard L, Elbrønd H, Laurberg S, Walker LR, Støvring J (2012). Danish national guidelines for treatment of diverticular disease. Dan Med J.

[4] Yoshida S, Hiyama K, Kirino I, Fukui Y, Terashima H (2022). Ascending colon stenosis caused by repeated diverticulitis that clinically mimicked advanced colon cancer: a case report. Int J Surg Case Rep.

[5] Chintapalli KN, Chopra S, Ghiatas AA, Esola CC, Fields SF, Dodd GD 3rd (1999). Diverticulitis versus colon cancer: differentiation with helical CT findings. Radiology.

[6] Nishiyama N, Mori H, Kobara H, Rafiq K, Fujihara S, Kobayashi M, Masaki T (2012). Difficulty in differentiating two cases of sigmoid stenosis by diverticulitis from cancer. World J Gastroenterol.

[7] Forshaw MJ, Sankararajah D, Stewart M, Parker MC (2006). Self-expanding metallic stents in the treatment of benign colorectal disease: indications and outcomes. Colorectal Dis.

[8] Ohta R, Sakon R, Goto M, Tachimori Y, Sekikawa K (2018). Self-expanding metal stent restenosis in obstructive colon diverticulitis mimicking colon cancer: a case report. Int J Surg Case Rep.

[9] Keränen I, Lepistö A, Udd M, Halttunen J, Kylänpää L (2010). Outcome of patients after endoluminal stent placement for benign colorectal obstruction. Scand J Gastroenterol.

[10] Tehranian S, Klinge M, Saul M, Morris M, Diergaarde B, Schoen RE (2020). Prevalence of colorectal cancer and advanced adenoma in patients with acute diverticulitis: implications for follow-up colonoscopy. Gastrointest Endosc.

